# Responses of Patients with Disorders of Consciousness to Habit Stimulation: A Quantitative EEG Study

**DOI:** 10.1007/s12264-018-0258-y

**Published:** 2018-07-17

**Authors:** Jingqi Li, Jiamin Shen, Shiqin Liu, Maelig Chauvel, Wenwei Yang, Jian Mei, Ling Lei, Li Wu, Jian Gao, Yong Yang

**Affiliations:** 1 Ming Zhou Nao Kang Rehabilitation Hospital, Hangzhou, 310000 China; 2 College of Life Information Science and Instrument Engineering, Hangzhou Dianzi University, Hangzhou, 310018 China; 3 Paris Descartes University, 45 Rue des Saints-Peres, 75006 Paris, France; 4 Rehabilitation Center, Wu Jing Hospital, Hangzhou, 310051 China

**Keywords:** EEG, Disorder of consciousness, Habit stimulation, Wavelet transformation, Nonlinear dynamics, Differential analysis

## Abstract

Whether habit stimulation is effective in DOC patient arousal has not been reported. In this paper, we analyzed the responses of DOC patients to habit stimulation. Nineteen DOC patients with alcohol consumption or smoking habits were recruited and 64-channel EEG signals were acquired both at the resting state and at three stimulation states. Wavelet transformation and nonlinear dynamics were used to extract the features of EEG signals and four brain lobes were selected to investigate the degree of EEG response to habit stimulation. Results showed that the highest degree of EEG response was from the call-name stimulation, followed by habit and music stimulations. Significant differences in EEG wavelet energy and response coefficient were found both between habit and music stimulation, and between habit and call-name stimulation. These findings prove that habit stimulation induces relatively more intense EEG responses in DOC patients than music stimulation, suggesting that it may be a relevant additional method for eliciting patient arousal.

## Introduction

Exploring an effective method to help the arousal of patients suffering from disorders of consciousness (DOCs) is an ongoing challenge [1, 2]. Sensitive stimulation treatments, including call-name and music stimulations, are the most commonly used methods [3, 4]. Habits such as alcohol consumption and smoking can arouse intense behavioral responses in normal individuals [5]. These habits are built on patients’ specific and unique lifestyle behaviors. The detailed neurobiological mechanisms associated with habits remain unclear. It is still unknown whether habit stimulation can be applied to DOC patients, as few studies have compared patient responses to habit with those to other types of stimulation [6].

Clinical practice indicates that habit stimulation (e.g. smoking) arouses stronger behavioral responses than non-habit stimulation (e.g. non-smoking) in DOC patients. For example, there was no response when vinegar or sauce was placed on the lips of patients who were alcoholics, but alcohol caused their lips to start moving in a drinking movement. Yet these behavioral responses may be subjective, and a quantitative study is needed to explain this phenomenon. In most cases, qualitative analyses such as neuropsychological scales [e.g. the Coma Recovery Scale – Revised (CRS-R) score] are used to evaluate brain functions in the clinical setting. In order to thoroughly investigate these behavioral responses, more quantitative analytical methods are needed to accurately evaluate the brain responses. Diagnostic tools such as functional magnetic resonance imaging [7–9], positron emission tomography [10, 11], and electroencephalogram (EEG) analysis [12–17] have been used as auxiliary diagnostic methods to evaluate the state of consciousness. EEG has several advantages over the other methods, including low cost, safety, easy access, and convenience for bedside evaluation [18–21].

Generally, linear and nonlinear dynamic analysis methods are used to extract the features of EEG signals. In linear analysis, one of the common methods is time-frequency analysis, including variable-time-interval Fourier transformation, wavelet transformation, and Wigner–Ville distribution. Among these, wavelet transformation is a typical time-frequency analysis with multi-resolution and multi-scale characteristics, which enables the conversion of a multi-signal from coarse to fine [22]. Wavelet transformation is a mature theory which has been widely used in the study of EEG signals [23]. Besides linear analysis, nonlinear dynamic analysis methods have also been widely used to extract features of EEG signals [23, 24]. Studies have shown that the human brain is a complex, multidimensional system, and the use of nonlinear dynamic analysis might accurately reflect brain states [25–27].

In this study, to investigate the response of DOC patients to habit stimulation, both linear and nonlinear dynamic analysis methods were applied to measure the response intensity of the EEG in DOC patients. In addition, topographic maps of the brain were plotted to evaluate the degree of response to stimulation in different regions.

## Materials and Methods

### Patients

A total of 19 patients with alcohol consumption or smoking habits were examined in this study, including 9 MCS (minimally conscious state) and 10 VS (vegetative state) cases assessed by neurologists based on the CRS-R. These patients were recruited from the Rehabilitation Center of Wu Jing Hospital in Zhejiang Province, China. Patients were out of the clinically acute phase, had been in a DOC state for > 1 month, exhibited spontaneous breathing, and had no history of cardiopulmonary resuscitation or neurological disease. The inclusion criteria were as follows: (a) inability to follow commands; (b) inability to clearly express words; (c) inability to open eyes even with stimulation or achieve eye tracking, not due to paralysis; (d) inability to move arms and legs in a non-directed fashion; and (e) having a Glasgow coma scale [28] score ≤ 8 points, and with a score for each item < 4-5-6-3-2-3 according to the CRS-R [29]. If the total score was ≥ 8, the patient was diagnosed as MCS; if it was < 8, the patient was diagnosed as VS. The exclusion criteria were as follows: (a) drug interventions (which could affect the assessment of brain function) prior to data collection such as nerve-muscle blockers, depressants, or anesthetics; and (b) a coexisting disorder such as metabolic disease, poisoning, or shock that could affect brain activity.

The state of consciousness was diagnosed by experienced clinicians from Wu Jing Hospital using the CRS-R scale. The regional Review Board approved the use of human participants in this study. Family members of patients and the attending doctor gave consent for EEG acquisition. Patient information is summarized in Table 1.

**Table 1:** Patient information.

Disease status	Number of patients	CRS-R score	Average age (years)	Time after injury (months)	Sex	
					Male	Female
MCS	9	13.7 ± 2.61	39.3 ± 11.9	3.10 ± 1.92	8	1
VS	10	6.2 ± 1.90	51.1 ± 10.2	4.05 ± 1.38	7	3

CRS-R, Coma Recovery Scale – Revised; MCS, minimally conscious state; VS, vegetative state.

### EEG Acquisition

EEG signals were recorded in single-electrode channel mode with an Active Two EEG system (BioSemi, Amsterdam, Netherlands). Electrodes were placed over the entire head according to the 10–20 general international standard lead system. Signals were recorded from 64 channels; the left and right earlobes were used as references.

The EEG recording was initiated when the signal had been stable for at least 2 min. Signals were digitized at a sampling rate of 256 Hz, a bandwidth range from 0.5 Hz to 70 Hz, and an electrode impedance < 5 KΩ.

**Fig. 1:**
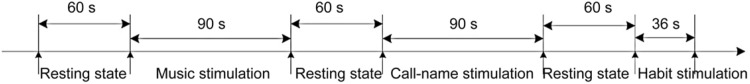
Time-course of EEG acquisition.

In the music stimulation, a piece of Chinese classical music “Jasmine” was truncated into voice fragments and played for 90 s. In the call-name stimulation, the patient’s name was called by relatives for 90 s. In the habit stimulation, patients were stimulated either by wiping alcohol on the lips for 36 s for alcoholic patients, or by introducing the smell of cigarette smoke for 36 s for smoking patients (Fig. 1). The entire process was repeated three times.

### EEG Signal Preprocessing

A time-window of 12 s was truncated from the acquired EEG data for analysis. IIR filter, an EEGLAB processing tool (University of California San Diego), was used to remove interference from the 50-Hz power frequency [30]. A wavelet soft threshold de-noising algorithm was applied to remove noise [31].

### Wavelet De-noising of EEG

Studies have shown that the process of wavelet de-noising often uses a given threshold for the de-noising paradigm. The most reliable threshold method is calculated based on trials and errors [31]. There are several classical threshold methods: (a) The VisuShrink threshold, also known as the general threshold, was the first wavelet threshold de-noising method developed. The probability that the coefficient is greater than the threshold is close to zero, so it is the optimal threshold method based on the minimum maximum estimation. (b) The Sureshrink threshold, also known as the Stein unbiased risk threshold, is close to the ideal threshold. This is an adaptive threshold selection based on Stein’s unbiased likelihood estimation criterion. (c) In the Heursure threshold, also known as the Heuristic Sure threshold, the threshold is chosen as the optimal predictor threshold, and is a synthesis of the first two thresholds. (d) The Minmax threshold uses a fixed threshold according to the minimum criteria to select the threshold.

In order to choose the most suitable threshold estimation method to analyze the data, Gaussian white noise was added to the original signal, and wavelet threshold de-noising and hard threshold de-noising were performed on the mixed signal. The VisuShrink, Sureshrink, Heursure, and Minmax methods were used for threshold estimation. The signal-to-noise ratio and the root-mean-square error were introduced as reference indicators (Table 2).

**Table 2:** Comparison of four threshold estimation methods.

Threshold estimation method	VisuShrink	Sureshrink	Heursure	Minmax
SNR	42.541	72.890	72.890	50.997
RSME	1.217	0.037	0.0370	0.460

SNR, signal-to-noise ratio; RSME, root-mean-square error.

The signal-to-noise ratio was maximal and the root-mean-square error was minimal in the processing methods of Sureshrink and Heursure threshold estimation. These results indicated that these two methods were equal and superior to the others. Therefore, both methods were used to analyze all our data.

### Wavelet Energy Extraction

**Fig. 2:**
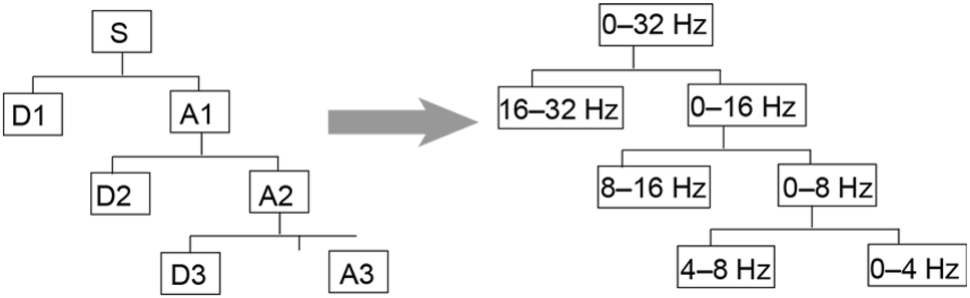
Correspondence of wavelet decomposition band diagram.

The signal was decomposed into eight layers, and a db3 wavelet base was chosen. As the eight-layer decomposition was complex, 0 Hz–32 Hz three-layer decomposition was taken as an example for the following wavelet decomposition corresponding band diagram (Fig. 2).

The energy value of a certain wavelet coefficient can be used to represent the energy value of a signal in a band. Since the wavelet function is an orthogonal basis function with energy conservation characteristics, the sum of the wavelet energy values of each band can represent the energy value of the signal, that is, the wavelet energy value of the signal:

$$ E_{n} = \sum\limits_{k} {\left| {d_{l}^{j,n} } \right|}^{2} $$

where both *n* and *k* are natural numbers and $d_{i}^{j,n}$are wavelet packet coefficients.

Wavelet transformation was used to calculate EEG signal features under resting and stimulation states (music, habit, and call-name). The resting state was considered as the unified normalized standard of response coefficients. Therefore, the ratio between the wavelet transform value of the stimulation state and the wavelet transform value of the resting state was defined as the wavelet energy value. This ratio reflected the change of wavelet energy before and after stimulation. The EEG wavelet energy was calculated as:

$$ Wavelet \, energy \, value \, \left( {ratio} \right)\; = \;wavelet \, energy \, value \, of \, stimulation \, state{ / }wavelet \, energy \, value \, of \, resting \, state. $$

### Nonlinear Dynamic Feature Extraction

Correlation dimension, complexity, entropy, and Lyapunov exponents are common nonlinear features in EEG signal analysis. Of these, entropy is the most suitable because of its small dataset demand and high computation speed, whereas correlation dimension and Lyapunov exponent require large datasets and strict dimensions (features that are unsuitable for EEG analysis). Therefore, in this study, approximate entropy (ApEn) [32], sample entropy (SampEn) [33], and permutation entropy (PmEn) [34], were used to determine the patients’ state of consciousness.

A nonlinear dynamic method was used to calculate EEG signal features (ApEn, SampEn, and PmEn) under the resting and stimulation states (music, habit, and call-name) [7]. During feature extraction, ApEn, SampEn, and PmEn were computed for the EEG data in each time-window (12 s signal truncated from the EEG data). For each nonlinear characteristic, the mean of the values from all time-windows was considered as a feature of the EEG data. The feature value was extracted and calculated from each channel. The means of single features were the average value of all 64 channel features of the EEG data.

The resting state was considered as the unified normalized standard of response coefficients (Rc). Therefore, the ratio between the nonlinear dynamic feature of the stimulation state and the nonlinear dynamic feature of the resting state was defined as the Rc. Rc values are the EEG nonlinear dynamic feature response coefficients of the stimulation value, which reflect the changes of a nonlinear dynamic feature before and after stimulation. The Rc values were calculated as:

$$ {\text{Rc}}\;{\text{value}}\;\left( {\text{ratio}} \right)\; = \;{\text{feature}}\;{\text{of}}\;{\text{stimulation}}\;{\text{state / feature}}\;{\text{of}}\;{\text{resting}}\;{\text{state}}. $$

RcA, RcS, and RcP refer to the Rc values for the three features ApEn, SampEn, and PmEn, respectively.

### Statistical Analysis

Wavelet energy and Rc values were analyzed using the paired-samples *t*-test, independent-sample *t*-test, and one-way ANOVA, using SPSS v.19 software (SPSS Inc., Chicago, IL). *P* < 0.05 was considered statistically significant.

## Results

### EEG Wavelet Energy Values for MCS and VS Under Different Stimulations

We first investigated the degree of EEG response to various kinds of stimulation by comparing the differences in wavelet energy. The highest wavelet energy of the cases (including MCS and VS) was for call-name stimulation, followed by habit and music stimulation (Table 3).

**Table 3:** Comparison of wavelet energy values in different stimulation states.

Stimulation	Total (MCS + VS)	MCS	VS
Music	1.124 ± 0.147	1.139 ± 0.158	1.107 ± 0.116
Habit	1.346 ± 0.198	1.391 ± 0.215	1.296 ± 0.115
Call-name	1.423 ± 0.314	1.489 ± 0.343	1.349 ± 0.128

**Fig. 3:**
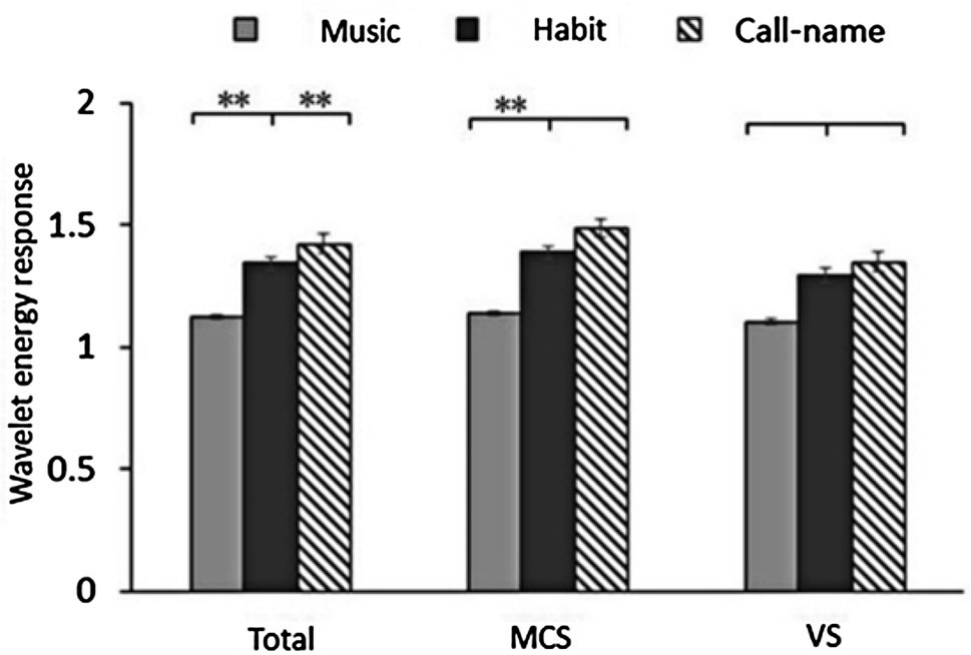
Difference of wavelet energy values in total cases (*n* = 19), MCS cases (*n* = 9), and VS cases (*n* = 10) in the three stimulations (music, habit, and call-name) (error bars, 95% confidence intervals; **P < 0.01).

To verify the differences among the three stimulations, statistical analysis of the wavelet energy values between the different stimulations were performed. First, differences were analyzed between habit and music stimulation, then between habit and call-name stimulation, using the paired-samples *t*-test. In all cases, there were significant differences in the wavelet energy both for habit *versus* music (P = 0.0065) and for habit *versus* call-name stimulation (*P* = 0.0089). In MCS cases, the wavelet energy differed significantly (*P* = 0.0074) between habit and music, but not between habit and call-name stimulation. However, the VS cases showed no significant differences in wavelet energy either between habit and music, or between habit and call-name stimulation (Fig. 3).

**Fig. 4:**
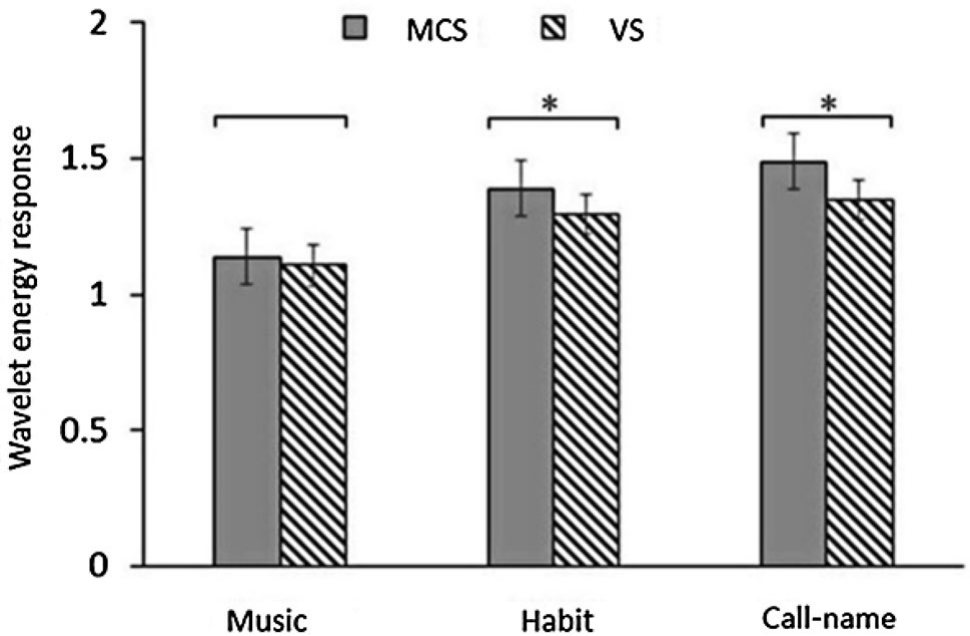
Analysis of EEG wavelet energy value differences between VS and MCS cases for the three stimulations (error bars, 95% confidence intervals; *P < 0.05).

Further investigations of the differences of wavelet energy values between VS and MCS cases for the three stimulations were performed using the independent-samples *t*-test. There was a significant difference in these values for habit (*P* = 0.023) and call-name stimulation (*P* = 0.016) between MCS and VS cases, but not for music stimulation (Fig. 4).

### EEG Rc Values for MCS and VS Under Different Stimulations

We then investigated the degree of EEG response to various kinds of stimulation by comparing the nonlinear entropy values in DOC patients. The Rc values (RcA, RcS, and RcP) of the three different stimulations are shown in Table 4. The results indicated that the highest Rc values among all the cases corresponded to call-name stimulation, followed by habit and music stimulation.

**Table 4:** EEG entropy values in different stimulation states.

Sample	Rc values		
	RcA	RcS	RcP
Total (MCS+VS)			
Music	1.044 ± 0.13	1.071 ± 0.12	1.004 ± 0.01
Habit	1.262 ± 0.05	1.234 ± 0.09	1.254 ± 0.31
Call-name	1.446 ± 0.24	1.254 ± 0.21	1.394 ± 0.32
MCS			
Music	1.025 ± 0.15	1.067 ± 0.15	1.004 ± 0.02
Habit	1.413 ± 0.07	1.377 ± 0.13	1.413 ± 0.23
Call-name	1.668 ± 0.21	1.644 ± 0.19	1.602 ± 0.28
VS			
Music	1.061 ± 0.13	1.074 ± 0.08	1.003 ± 0.004
Habit	1.127 ± 0.09	1.105 ± 0.06	1.111 ± 0.36
Call-name	1.247 ± 0.17	1.211 ± 0.15	1.206 ± 0.24

**Fig. 5:**
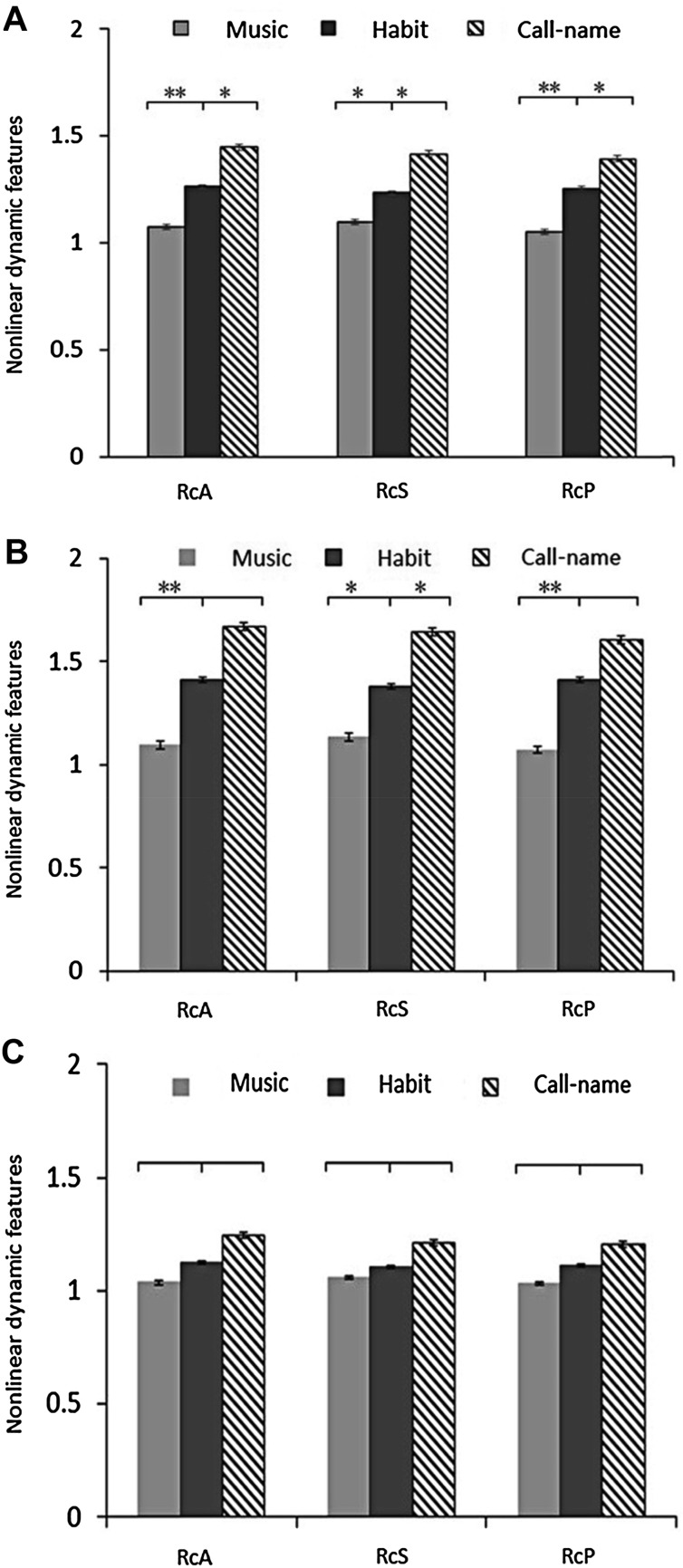
Rc values of **A** the total patient population (*n* = 19), **B** MCS cases (*n* = 9), and **C** VS cases (*n* = 10) in response to the three stimulations (error bars, 95% confidence intervals; *P < 0.05, **P < 0.01).

In order to verify the statistical differences between the three stimulations, statistical analysis of the Rc values for habit *versus* music, and for habit *versus* call-name stimulation was performed using the paired-samples *t*-test. In all cases, there were significant differences in the three Rc values between habit *versus* call-name stimulation (RcA, *P* = 0.029; RcS, *P* = 0.034; RcP, *P* = 0.041). Moreover, two Rc values (RcA and RcP) remarkably differed between habit and music stimulations (*P* = 0.0082, *P* = 0.0096) (Fig. 5A). In MCS cases, there were significant differences in the three Rc values between habit and music stimulations (RcA, *P* = 0.0097; RcS, *P* = 0.037; RcP, *P* = 0.0088); but only RcS differed (*P* = 0.038) between habit and call-name stimulations (Fig. 5B). In VS cases, there were no differences in the three Rc values either between habit and music or between habit and call-name stimulations (Fig. 5C).

Further comparison of the Rc values between VS and MCS cases was performed using the independent-samples *t*-test. RcA and RcS differed significantly (RcA, *P* = 0.006; RcS, *P* = 0.004) between MCS and VS cases for habit stimulation; all three differed significantly (RcA, *P* = 0.007; RcS, *P* = 0.001; RcP, *P* = 0.003) between MCS and VS cases for call-name stimulation; and none was significantly different between MCS and VS cases for music stimulation (Table 5).

**Table 5:** Analysis of EEG Rc differences (*P* values) between VS and MCS for the three stimulations.

Feature value	Music	Habit	Call-name
RcA	0.177	0.006	0.007
RcS	0.894	0.004	0.001
RcP	0.871	0.032	0.003

### EEG Response in Different Brain Lobes Under Different Stimulations

Since different stimulations may activate distinct brain areas, we finally investigated the degree of EEG responses in the frontal, temporal, parietal, and occipital lobes at different regions elicited by various types of stimulation.

Among the nonlinear EEG features (ApEn, SampEn, and PmEn), PmEn measures the complexity of a signal, with the advantages of being simple, fast, and having a strong anti-noise property. Therefore, PmEn was used to measure the nonlinear dynamic feature responses of the different areas. The differences of entropy values in the different areas during the three stimulations were evaluated using one-way ANOVA.

The resulting EEG responses in the different lobes under different stimulations are shown in Table 6. During habit stimulation, the RcP values in the frontal lobe were significantly higher than those in any other areas in VS cases (*P* < 0.05), MCS cases (*P* < 0.01), and VS + MCS cases (*P* < 0.01). During music stimulation, the RcP values in the temporal lobe were significantly higher (*P* < 0.05) than those in any other areas for VS + MCS and VS cases, but not for MCS cases. During call-name stimulation, the RcP values in the temporal lobe were significantly higher than those in any other areas for VS + MCS (*P* < 0.05), VS (*P* < 0.05), and MCS (*P* < 0.01).

**Table 6:** EEG entropy values in different stimulation states.

Sample	Brain area			
	Frontal lobe	Temporal lobe	Parietal lobe	Occipital lobe
Total (MCS+VS)				
Music	0.986 ± 0.097	1.048 ± 0.105*	1.003 ± 0.079	0.985 ± 0.103
Habit	1.452 ± 0.132**	1.116 ± 0.097	1.179 ± 0.099	1.294 ± 0.154
Call-name	1.378 ± 0.112	1.530 ± 0.101*	1.315 ± 0.101	1.393 ± 0.145
MCS				
Music	1.014 ± 0.059	1.024 ± 0.062	1.005 ± 0.050	0.972 ± 0.057
Habit	1.664 ± 0.163**	1.215 ± 0.099	1.280 ± 0.104	1.492 ± 0.138
Call-name	1.601 ± 0.068	1.730 ± 0.110**	1.447 ± 0.126	1.631 ± 0.162
VS				
Music	0.973 ± 0.122	1.072 ± 0.135*	0.967 ± 0.101	0.998 ± 0.135
Habit	1.240 ± 0.103*	1.018 ± 0.100	1.077 ± 0.099	1.096 ± 0.165
Call-name	1.155 ± 0.139	1.331 ± 0.092*	1.183 ± 0.064	1.154 ± 0.099

*P < 0.05, **P < 0.01.

**Fig. 6:**
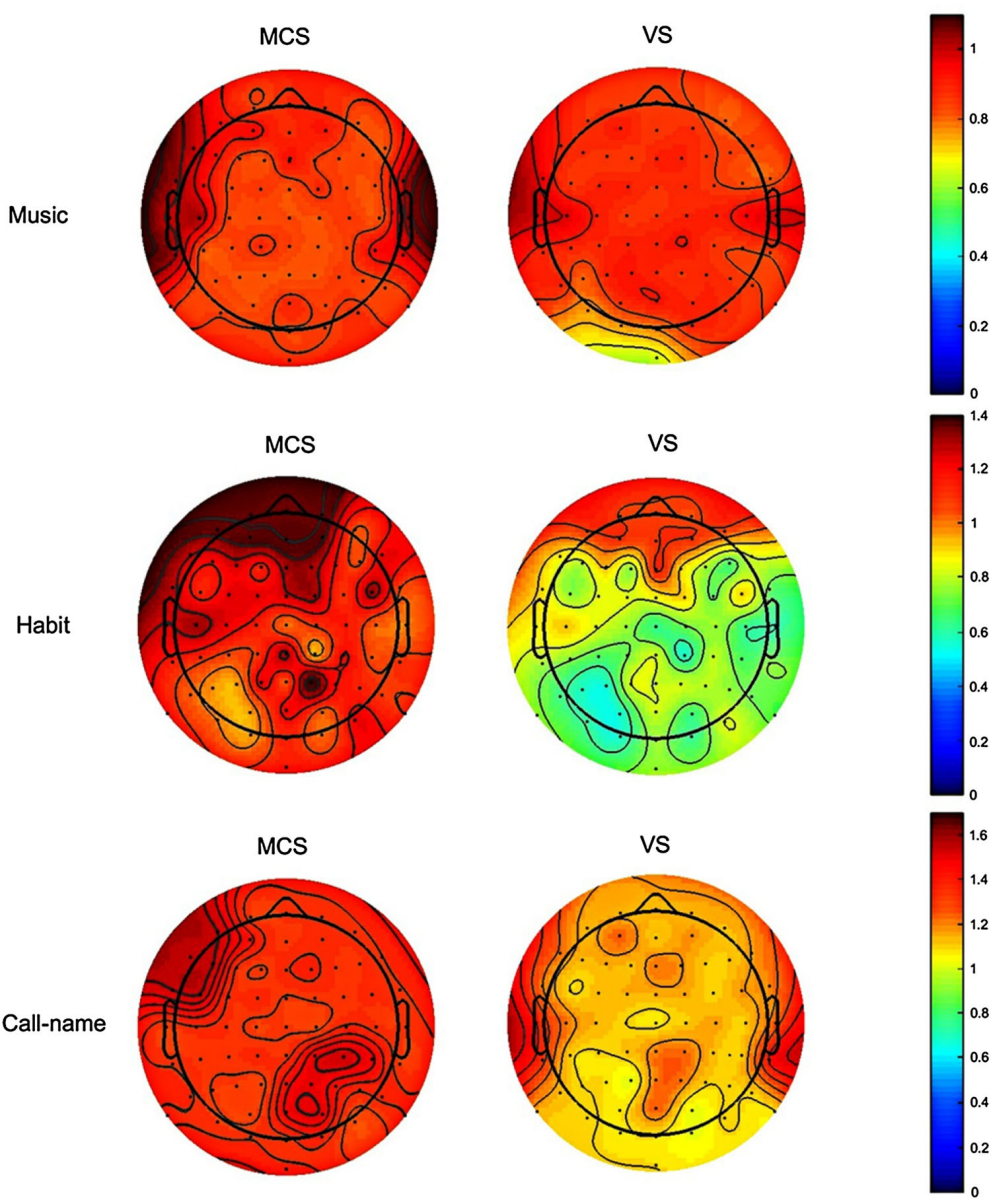
EEG topographic maps of the mean PmEn under the three stimulations. The colors reflect the intensity of the EEG response. MCS and VS results for music stimulation (upper panels), habit stimulation (middle panels), and call-name stimulation (lower panels).

To visualize the results of brain responses under different stimulations, we used the topographic map visualization method. Three VS cases and 4 MCS cases were chosen to plot the maps using the mean PmEn (Fig. 6). These patients did not suffer from traumatic brain injury, but from other conditions such as subarachnoid hemorrhage or diffuse axonal injury. Indeed, in these cases, the brain integrity was conserved and the mapping was not influenced by injury of the lobes.

With the music stimulation, in both MCS and VS cases, the darkest area was located in the temporal cortex, denoting a significantly more intense EEG response than in other regions. Furthermore, the EEG responses in MCS cases were more intense than those of VS cases. The results of music stimulation showed that, in the selected cases, the temporal region was the most responsive.

With the habit stimulation, in both MCS and VS cases, the darkest area was located in the prefrontal region, which had significantly more intense EEG responses than other regions. The EEG responses of the MCS cases were more intense than those of the VS cases. The results of habit stimulation demonstrated that, for the selected cases, the frontal brain region was the most responsive area.

With the call-name stimulation, in the MCS cases, the darkest areas were in the left temporal and right occipital areas, which had significantly more intense EEG responses than other regions. In the VS cases with call-name stimulation, the darkest area was in the temporal area, which had significantly more intense EEG responses than other regions. The EEG responses of MCS cases were more intense than those of VS cases.

## Discussion

In this study, using EEG wavelet transformation and nonlinear dynamics analysis, we studied the EEG responses of DOC patients under different stimulations to determine whether habit stimulation is clinically useful. We found that, with the three types of stimulation (music, habit, and call-name), the EEG response under habit stimulation was higher than that under music stimulation, but lower than that under call-name stimulation. In different consciousness states, the MCS response to habit stimulation was more pronounced than that for VS patients. These results provide preliminary evidence for the effectiveness of habit stimulation. Concerning the spatial distribution of EEG responses, the brain maps showed a more intense response in the frontal lobe during habit stimulation and in the temporal lobe during music and call-name stimulation. These results may reflect positive neural-related activity evoked by the stimulations.

Results from current studies suggest that habit stimulation can arouse brain responses; however, the detailed neurobiological mechanism underlying habit remains unclear but could be involved in the addiction mechanism. Existing research shows that the mesolimbic dopamine system (MLDS) is the neurobiological basis of the addiction mechanism [35]. The MLDS is a pathway by which dopamine is carried from one area to another. Dopamine is the major molecule released by the brain’s reward centers. Dopaminergic neurons are mainly located in the ventral tegmental area, and also have projections into several parts of the brain, including the nucleus accumbens, prefrontal cortex, hippocampi, and amygdala. The MLDS is the common neural pathway of the reward mechanism, and is involved in the physiological process of addiction [36]. In nicotine habit stimulation, the MLDS is thought to be the key site of its action. However, nicotine also increases the extracellular dopamine concentration by stimulating dopaminergic neurons. In alcohol habit stimulation, it has been reported that alcohol stimulates addiction, triggers dopamine release, enhances brain activity, and stimulates the reward system. Rose [37] proposed that nicotine and alcohol have positive reinforcement in the MLDS. The specific neural mechanisms involved in habit stimulation need to be further clarified using imaging techniques such as fMRI combined with EEG, as well as neuropsychological scale methods. Future investigations need larger samples.
